# The utility of a differentiated preclinical liver model, HepaRG cells, in investigating delayed toxicity via inhibition of mitochondrial-replication induced by fialuridine

**DOI:** 10.1016/j.taap.2020.115163

**Published:** 2020-09-15

**Authors:** Carol E. Jolly, Oisin Douglas, Laleh Kamalian, Rosalind E. Jenkins, Alison J. Beckett, Sophie L. Penman, Dominic P. Williams, Mario Monshouwer, Damir Simic, Jan Snoeys, B. Kevin Park, Amy E. Chadwick

**Affiliations:** a MRC Centre for Drug Safety Science, The Department of Pharmacology and Therapeutics, The University of Liverpool, Ashton Street, Liverpool L69 3GE, UK; b Cellular and Molecular Physiology, The University of Liverpool, Crown Street, Liverpool L69 3BX, UK; c Innovative Medicines and Early Development, Drug Safety and Metabolism, Translational Safety, AstraZeneca, Cambridge, UK; d Pharmacokinetics Dynamics and Metabolism, Janssen Research and Development, Beerse, Belgium; e Mechanistic and Investigative Toxicology, Janssen Research and Development, Spring House, PA, USA

**Keywords:** ALR, ATP-linked respiration, BR, basal respiration, CE, coupling efficiency, Cmax, maximal plasma concentration, DILI, drug-induced liver injury, FIAU, fialuridine, FIAM, pharmacologically inactive epimer of fialuridine, hENT1, human equilibrative nucleoside transporter 1, MR, maximal respiration, mtDNA, mitochondrial DNA, PHH, primary human hepatocytes, PL, proton leak, SRC, spare respiratory capacity, TEM, transmission electron microscopy, TK1, TK2, thymidine kinase-1 and -2, Fialuridine, HepaRG, Mitochondria, mtDNA, Hepatotoxicity, Liver

## Abstract

During its clinical development fialuridine caused liver toxicity and the death of five patients. This case remains relevant due to the continued development of mechanistically-related compounds against a back-drop of simple in vitro models which remain limited for the preclinical detection of such delayed toxicity. Here, proteomic investigation of a differentiated, HepaRG, and proliferating, HepG2 cell model was utilised to confirm the presence of the hENT1 transporter, thymidine kinase-1 and -2 (TK1, TK2) and thymidylate kinase, all essential in order to reproduce the cellular activation and disposition of fialuridine in the clinic. Acute metabolic modification assays could only identify mitochondrial toxicity in HepaRG cells following extended dosing, 2 weeks. Toxic effects were observed around 10 μM, which is within a range of 10–15 X approximate Cmax. HepaRG cell death was accompanied by a significant decrease in mitochondrial DNA content, indicative of inhibition of mitochondrial replication, and a subsequent reduction in mitochondrial respiration and the activity of mitochondrial respiratory complexes, not replicated in HepG2 cells. The structural epimer of fialuridine, included as a pharmacological negative control, was shown to have no cytotoxic effects in HepaRG cells up to 4 weeks. Overall, these comparative studies demonstrate the HepaRG model has translational relevance for fialuridine toxicity and therefore may have potential in investigating the inhibition of mitochondrial replication over prolonged exposure for other toxicants.

## Highlights

• Metabolic modification has been adapted to identify delayed (weeks) mitochondrial dysfunction.
• Fialuridine induces delayed mitochondrial dysfunction and toxicity at physiologically relevant exposures.
• Inhibition of mitochondrial replication leads to decreased oxidative phosphorylation before cell death.
• HepaRG cells are a more suitable model for fialuridine-induced toxicity than HepG2 cells.
• HepaRG cells offer a practical model to study toxicity via effects on mitochondrial replication.

## 1 Introduction

The failed fialuridine (FIAU) clinical trial is infamous for the severity of the adverse effects, which resulted in five deaths from acute liver failure, with a further two patients only surviving following liver transplantation (McKenzie et al., 1995). This outcome was unexpected; the drug had been part of four previous clinical trials in which no toxicity had been identified. However, it was the significant extension of the clinical trial period that ultimately led to toxicity, with effects only becoming apparent after week 13 of treatment. Despite having never reached the market, FIAU remains a unique compound in the field of drug-induced liver injury (DILI), not just because of its unexpected and severe nature, but because it is the clearest example of direct drug-induced mitochondrial toxicity as the causative event in hepatotoxicity and its extreme species-selective toxicity (McKenzie et al., 1995; Lewis et al., 1996). Thus, FIAU continues to be a valuable paradigm compound in the field of DILI research, particularly in the development of improved models for the prediction of delayed DILI and also mitochondrial toxicity induced by nucleotide-based antiviral therapy. Moreover, current relevance is enhanced by the continued development of antiretroviral drugs for which preclinical evaluation of mitochondrial dysfunction is recommended by the Food and Drug Administration of the U.S. Government (FDA, 2015). The purpose of this work is therefore to characterise the use of HepaRG cells as a practical model with clinical relevance suitable for the preclinical evaluation of FIAU induced toxicity.

After the clinical trial was halted, histopathological analysis revealed evidence of hepatic steatosis and abnormal mitochondria within the damaged livers suggesting that FIAU-toxicity was mediated via mitochondrial dysfunction (McKenzie et al., 1995). Lactic acidosis was also apparent as the first sign of toxicity, consistent with electron transport chain dysfunction (Rossignol et al., 2003; Robinson, 2006; Andersen et al., 2013). Subsequently, it was demonstrated that the target of FIAU is mitochondrial DNA (mtDNA), an effect arising in part from FIAU's structure as a nucleoside analogue (Lewis et al., 1996; Horn et al., 1997). Once phosphorylated to the triphosphate form FIAU can inhibit mtDNA replication via its insertion in to the growing chain, with the eventual presence of several consecutive FIAU units leading to the inability of mitochondrial DNA polymerase γ to replicate the DNA (Lewis et al., 1996). It is now understood that the lack of toxicity in in vivo models arises due to the species selective uptake of FIAU into the mitochondria via the human equilibrative nucleoside transporter-1 (hENT-1, SLC29A1) (Lai et al., 2004; Xu et al., 2014). Based upon this knowledge, the key cellular characteristics that must be present in a model in order to recapitulate the clinical aetiology of FIAU-induced hepatotoxicity, include the presence of the hENT1 transporter, active mitochondrial thymidine kinases, and the ability to perform extended toxicological studies in order to mirror the delayed toxicity in patients. Historically, in vitro investigations of the effects FIAU on mtDNA have been performed predominantly in proliferating HepG2 cells over acute periods (Colacino et al., 1994; Lewis et al., 1996; Birkus et al., 2003). Therefore, there is a need to characterise an in vitro model which retains practicality and reproducibility but with improved human physiological relevance which can be used over longer periods with lower FIAU concentrations, to thus allow FIAU-induced effects on the mitochondria to accumulate.

**Fig. 1:**
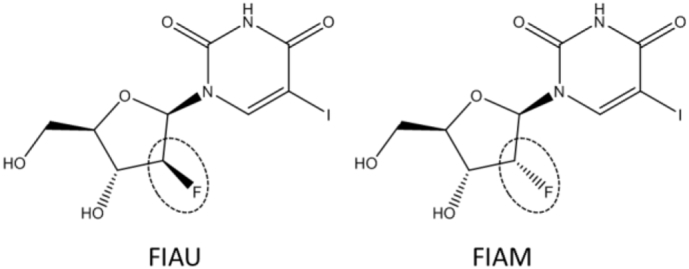
The structures of fialuridine (FIAU) and its stereoisomer, the epimer (FIAM). In FIAU the fluorine atom is in the S configuration, whereas in FIAM fluorine it is in the R configuration, as indicated.

HepaRG cells were originally generated from a hepatic carcinoma and are a mixture of hepatocyte-like and biliary-like epithelial cells which are closer in characteristics to primary human hepatocytes than HepG2 cells. In their differentiated form they can be kept in culture for up to 4 weeks, and are polarised with evidence of biliary structures (Gerets et al., 2012). Here we use a strategy, employed and described by us previously, in which the physiological and pharmacological relevance of an in vitro model is confirmed to define whether it is fit for purpose before embarking on toxicological studies (Kamalian et al., 2015; Kamalian et al., 2018; Weaver et al., 2019). This is particularly important due to the known differences in key transporters and enzymes of drug disposition, for example phase I and phase II metabolism enzymes, across in vitro models and primary human hepatocytes (Sison-Young et al., 2015; Bell et al., 2016). Therefore, firstly, proteomic confirmation of the presence of critical proteins required for FIAU phosphorylation and mitochondrial uptake was performed. Next we expanded our previous use of HepaRG cells to detect mitochondrial toxicants by acute exposure (Kamalian et al., 2018) and here describe the use of HepaRG cells as a model for FIAU toxicity over extended exposure, including the first description of the use of the metabolic modification screen for mitochondrial toxicity over an extended period of weeks. Finally, the mitotoxicological effect was examined over a 4-week period assessing mtDNA content, mitochondria respiratory function, glycolytic function and cell viability. The pharmacologically inactive epimer of FIAU, FIAM, in which the fluorine molecule has a different conformation, was included as a negative chemical control (Fig. 1). This difference in chiral structure results in a compound which is inactive pharmacologically and toxicologically. Importantly, throughout, model suitability was compared to HepG2 cells.

## 2 Methods and materials

### 2.1 Materials

Foetal bovine serum (FBS), dialysed foetal bovine serum (DFBS) and PCR reagents (Applied Biosystems) were obtained from ThermoFisher Scientific (Paisley, UK). Plateable cryopreserved hepatocytes were purchased from KaLy Cell (Plobsheim, France). HepaRG cells, media and supplements were purchased from Biopredic International (Saint Grégoire, France). HepG2 cells came from ECACC general cell collection were purchased from Public Health England (Salisbury, UK). All Seahorse consumables were purchased from Agilent Technologies (Cheshire, U.K.). The QIAamp DNA Mini Kit was purchased from Qiagen (Crawley, UK), and all other PCR reagents were purchased form Applied Biosystems (California, USA). The L-lactate assay kit was purchased from ScienCell (California, USA), with the remaining materials and reagents were purchased from Sigma-Aldrich (Poole, Dorset, UK).

### 2.2 Cell culture

In accordance with Biopredic international guidelines, undifferentiated HepaRG cells were seeded into clear flat bottomed 96 well (9000 cells/well) or Seahorse plates (4000 cells/well). They were grown for two weeks in growth media before their differentiation over 2 further weeks in differentiation media. Cells were kept at 37 °C, 5% CO_2_ in a humidified environment and received fresh, appropriate media twice a week. The fully differentiated cells were then used over the following four-week period, whilst being maintained in differentiation media. All experiments were performed using cells at passage number < 20.

HepG2 cells were cultured and maintained in DMEM media as described previously (Kamalian et al., 2015).

### 2.3 iTRAQ analysis

The experimental comparison of the proteome of HepaRG cells and cryopreserved primary human hepatocytes (PHH) was performed and reported in a previous publication (Sison-Young et al., 2015). Briefly, three biological replicates of HepaRG and HepG2 cells were compared to cryopreserved PHH from 3 different donors across 3 iTRAQ experiments, with a common master pool in each. The proteomic data deposited from the previous study was mined, using ProteinPilot (version 5.0, Sciex) in order to quantify the relative expression level of four proteins that are critical in the development of FIAU-induced mitochondrial toxicity. The entire dataset (2867 proteins with quantitative data in every sample) was analysed by 2-way ANOVA (Partek Genomics Suite) with cell type and iTRAQ experiment as factors.

### 2.4 Fialuridine dosing protocol

Differentiated HepaRG cells were treated with 0–300 μM final concentrations of FIAU (1–4 weeks). FIAU was replaced in fresh differentiation media every 3–4 days. The final concentration of DMSO was at a constant 0.5% *v*/v in both HepaRG and HepG2 treatments. In all experiments dosing was performed in glucose supplemented media.

### 2.5 Metabolic modification assay: Dual ATP and cell viability assessment

This was performed as previously described (Kamalian et al., 2018). Briefly, following the dosing period, media was switched to either “glucose” or “galactose” media for 2 h prior to measuring cellular ATP content and lactate dehydrogenase (LDH) release as a marker of cell viability, both normalised to protein content. IC_50_ values were calculated using Prism (GraphPad, California, USA) (Table 1) and criteria applied to identify a compound as a direct mitochondrial toxin.

### 2.6 Seahorse XFe analysis of oxygen consumption rates

Briefly, following the dosing period, media was switched to unbuffered XF basal medium (175 μl, pH 7.4) supplemented with glucose (25 mM), l-glutamine (2 mM) and sodium pyruvate (1 mM) before incubating in a CO_2_ free incubator (37 °C). Oxygen consumption was measured by Seahorse XFe96 according to the previously described protocol (Kamalian et al., 2018).

The assessment of individual mitochondrial complex activity was preformed using the in-situ mito substrate assay in permeabilised cells using Seahorse Technology as previously described (Ball et al., 2016).

### 2.7 Determination of cellular nuclear and mitochondrial DNA content using RT-PCR

DNA was extracted via QIAamp DNA Mini Kit (Qiagen, Crawley, UK). Relative levels of mtDNA copy number were determined by real-time pre-designed PCR Taqman detection kits designed to target the mitochondrial MT-ND1 gene and the nuclear RNase P gene, using TAQMAN GT Master Mix. PCR reactions were performed according to standard conditions for TaqMan: 95 °C for 10 min; 40 cycles at (95 °C 15 s, 60 °C 1 min). PCR assays were performed in duplicate for each DNA sample. Analysis was undertaken with mitochondrial ND-1 normalised to genomic RNase P and treated sample to the appropriate vehicle.

### 2.8 Determination of cellular steatosis using Oil Red O Staining

Following drug treatment cells were washed twice in PBS and fixed in 4% paraformaldehyde (1 h, RT). Cells were then washed twice in ddH_2_O and incubated in isopropanol (IPA, 60% *v*/v, 5 min). Oil red O working solution (3:2 in ddH_2_O from the stock solution of 0.33% *w*/*v* in 100% IPA) was applied to the cells for 20 min. Background staining was removed by several washes with ddH_2_O and a 5 min wash in 60% v/v IPA. The intracellular lipid was extracted by 100% IPA after the wells were dried. The lipid content was quantified by reading the optical density at 540 nm on a Varioskan Flash (Thermo Fisher Scientific, USA).

### 2.9 Determination of cellular L-lactate release

Following incubation with drug the media from cells was transferred to a fresh 96-well plate and L-lactate levels were measured using an L-lactate assay kit as per the manufacturer's instructions.

### 2.10 Electron microscopy imaging

Samples were prepared for transmission electron microscopy (TEM) in 3 cm dishes as follows. Cells were fixed in 2.5% (*w*/*v*) glutaraldehyde in 0.1 M phosphate buffer (pH 7.4) before staining with reduced osmium (2% (w/v) OsO4, 1.5% (w/v) potassium ferrocyanide in ddH_2_O), 1% (w/v) thiocarbohydrazide, 2% OsO4 (w/v in ddH_2_0), then 1% (w/v) aqueous uranyl acetate overnight at 4 °C. The next day samples were finally stained with Walton's lead aspartate (0.02 M lead nitrate, 0.03 M aspartic acid, pH 5.5). To prevent precipitation artefacts the cells were washed copiously with ddH_2_O between each staining step. Unless stated, fixation and staining steps were performed in a Pelco Biowave®Pro (Ted Pella Inc. Redding California, USA) at 100 w 20 Hg, for 3 mins and 1 min respectively. Dehydration was in a graded ethanol series before filtration and embedding in medium premix resin (TAAB, Reading, UK).

For TEM, 70–74 nm serial sections were cut using a UC6 ultra microtome (Leica Microsystems, Wetzlar, Germany) and collected on Formvar (0.25% (w/v) in chloroform, TAAB, Reading, UK) coated Gilder 200 mesh copper grids (GG017/C, TAAB, Reading, UK). Images were acquired on a 120 kV Tecnai G2 Spirit BioTWIN (FEI, Hillsboro, Oregon, USA) using a MegaView III camera and analySIS software (Olympus, Germany).

It should be noted that due to the practicalities of experimental set-up, over the 8-week period of differentiation followed by drug exposure, sample processing was not optimal and it was seen that cell dissociation occurred during preparation for TEM. Therefore, TEM images do not represent differentiated HepaRG morphology, which was maintained throughout treatment, see Fig Supp 1. Resultant TEM images were analysed using Image J software to measure intracellular mitochondrial features (NIH, USA). All processing and analysis were performed blinded. In brief, total mitochondria number per cell was counted as well as mitochondria area (μM^2^) alongside cytosol area (calculated as total cell area minus nuclear area and vacuole area μM^2^). For each treatment, at least 100 cells were analysed with results presented as the subsequent average of all cells that were analysed.

### 2.11 Statistical analysis

Each experiment was performed on three or more independent occasions (*n* ≥ 3). IC_50_ values were calculated using GraphPad Prism 7 software, and were expressed as a mean ± standard deviation (SD). For all other statistical analysis comparing the values obtained from treated cells to vehicle control an ANOVA was performed, with a Dunnett or Tukey post hoc analysis where appropriate. *P* values lower than 0.05 were considered as statistically significant for both tests.

## 3 Results

### 3.1 HepaRG cells possess the transporters and enzymes required for the phosphorylation of fialuridine in the mitochondria

Comparative proteomic analysis of the HepaRG and HepG2 cells was performed using iTRAQ methods in order to quantitatively assess the phenotypic similarities and differences with cryopreserved primary human hepatocytes (PHH) which were analysed after overnight cultivation in a plated format (Sison-Young et al., 2015). It was postulated that this database could provide a rational basis for the context-specific selection of the most appropriate ‘hepatocyte-like’ cell for the evaluation of particular cellular functions associated with DILI, e.g. delayed mitochondrial toxicity. This data was mined to confirm the presence of several enzymes and transporters that are critical in the toxicity of FIAU; hENT-1, thymidine kinase-1 and -2 (TK1, TK2) and thymidylate kinase were identified in the samples, with MS/MS evidence and protein sequence coverage presented in Fig Supp 2. Analysis of the quantitative data revealed that all four proteins were present in HepaRG cells, although hENT-1 was present at significantly lower levels compared with PHH (Table 1). The level of thymidylate kinase was significantly raised in HepaRG cells relative to PHH, though only a 1.39-fold-increase was observed. There was no significant difference in the level of TK-1 and TK-2 between HepaRG cells and PHH. It is notable that, apart from hENT1, the three remaining proteins are present at significantly greater levels in HepG2 than PHH. However, it is clear that there is a large variation in protein levels within PHH, which may arise due to inter-individual differences across populations. hENT-1, TK1 and TK2 are all expressed at low levels and thymidylate kinase at medium level in liver in vivo (Human Protein Atlas, www.proteinatlas.org, (Uhlen et al., 2015) and this is reflected in the small number of peptides on which the quantitative analysis is based. However, the observed changes were generally consistent across three independent HepaRG/HepG2 samples.

**Table 1:** The proteome of HepaRG and HepG2 cells was compared to that of cryopreserved human hepatocytes from 3 donors using iTRAQ analysis. Three biological replicates of HepaRG cells, HepG2 and cryopreserved human hepatocytes (from 3 different donors) were compared across 3 iTRAQ separate experiments. Relative quantification data for proteins involved in mitochondrial uptake of fialuridine was extracted from the complete dataset. The number of peptides at 99% confidence refers to the number used for quantification rather than identification. NQ – not quantified.

	iTRAQ1				iTRAQ2				iTRAQ3				HepaRG vs PHH	
Protein Accession no.	No peptides (99% conf)	HepaRG1: PHH1	HepaRG2: PHH2	HepaRG3: PHH3	No peptides (99% conf)	HepaRG1: PH14	HepaRG2: PHH2	HepaRG3: PHH3	No peptides (99% conf)	HepaRG1: PHH1	HepaRG2: PHH2	HepaRG3: PHH3	*p*-value	Fold change
hENT-1 Q99808	2	0.395	0.608	0.718	1	0.055	0.081	0.103	1	0.020	0.027	0.028	0.0001	−9.17
Thymidylate kinase P23919	2	1.236	1.660	1.306	3	1.432	2.032	1.803	3	0.929	1.159	1.180	0.015	1.39
TK-1 P04183	1	0.363	0.366	0.895	2	0.171	0.161	0.147	0	NQ	NQ	NQ	0.555	−1.28
TK-2 O00142	1	0.809	0.745	0.824	1	0.520	0.550	0.809	2	0.817	1.236	0.863	0.289	−1.28


	iTRAQ1				iTRAQ2				iTRAQ3				HepG2 vs PHH	
Protein Accession no.	No peptides (99% conf)	HepG21: PHH1	HepG22: PHH2	HepG23: PHH3	No peptides (99% conf)	HepG21: PHH1	HepG22: PHH2	HepG23: PHH3	No peptides (99% conf)	HepG21: PHH1	HepG22: PHH2	HepG23: PHH3	p-value	Fold change
hENT-1 Q99808	2	1.820	2.805	3.311	1	0.920	2.700	2.535	1	0.991	1.675	1.599	0.15	1.89
Thymidylate kinase P23919	2	1.445	1.977	1.542	3	2.291	3.251	2.831	3	8.709	10.092	25.586	0.0002	1.83
TK-1 P04183	1	4.613	4.285	12.246	2	9.818	4.207	4.093	0	NQ	NQ	NQ	0.00009	9.28
TK-2 O00142	1	0.575	0.525	0.586	1	0.268	0.286	0.417	2	0.752	1.127	0.794	0.0165	0.54

### 3.2 An extended time-course reveals that fialuridine induced cell death via mitochondrial dysfunction after 14 days

**Fig. 2:**
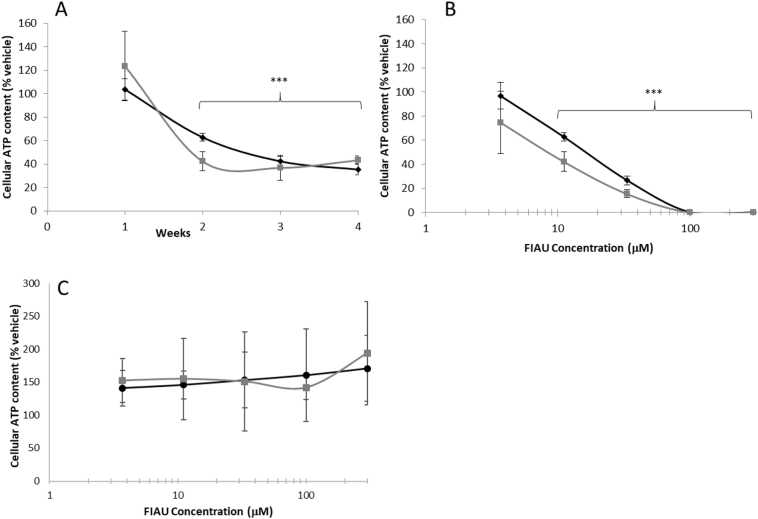
Cellular ATP content after metabolic modification assay performed in HepaRG and HepG2 cells exposed to FIAU (0–300 μM). Cellular ATP content (ATP/μg protein) is reported as a percentage of the vehicle control in both glucose and galactose media. A: Time-response curve following exposure of HepaRG cells to 11 μM FIAU. B: Concentration-response curve following 2 weeks exposure of HepaRG cells. C: Concentration-response curve following 72 h exposure of HepG2 cells. Key: Round black markers represent data collected in glucose media, square black markers represent data collected in galactose media. Key: Significance of each data point compared to vehicle control; *** *p* < .001, *n* = 3, error bars represent standard deviation.

**Fig. 3:**
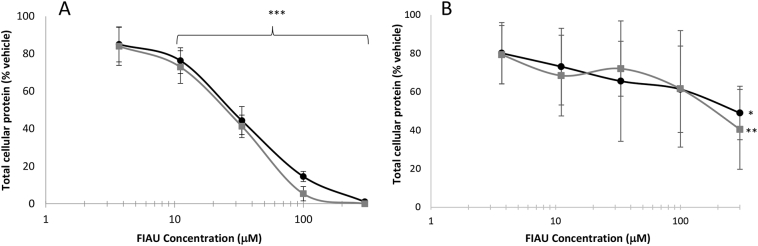
Total cellular protein after metabolic modification assay performed in HepaRG and HepG2 cells exposed to FIAU (0–300 μM). Total cellular protein (μg) is reported as a percentage of the vehicle control in both glucose and galactose media. A: Concentration-response curve following 2 weeks exposure of HepaRG cells. B: Concentration-response curve following 72 h exposure of HepG2 cells. Key: Round black markers represent data collected in glucose media, grey square markers represent data collected in galactose media. Significance of each data point compared to vehicle control; * *p* < .05, ** *p* < .01), n = 3, error bars represent standard deviation.

The acute metabolic modification method was used to examine cytotoxicity induced by FIAU over a 4-week period. Dosing was performed in normal growth media with glucose as the sugar source. Metabolic modification is established by replacing media to either glucose or galactose media for the final 2 h of incubation. Mitochondrial pertubations were defined based upon the criteria previously described (Kamalian et al., 2015). In differentiated HepaRG cells FIAU was seen to induce a concentration- and time- dependent decrease in cellular ATP content in both glucose and galactose media (Fig. 2A, B). Significant differences in cellular ATP content were apparent when cells were assayed in galactose media over glucose media at week 2, whilst this difference between the media was not seen at weeks 3, or 4, ATP levels were decreased equally. A concomitant time-dependent decrease in total cellular protein were also evident over the exposure period (Fig. 3A), which due to the differentiated status of HepaRG cells can be viewed as a measure of cell viability. Importantly, the reduction in ATP at week 2 (11 μM, Fig. 2A) occurred prior to a loss in cellular protein (Fig. 3A) which exemplifies the point at which mitochondrial respiration is perturbed before cell viability is decreased. The lack of difference in ATP levels between glucose and galactose media at later weeks occurs in tandem with a decrease in cell health and therefore is not indicative of solely mitochondrial dysfunction.

The effects of FIAU on HepG2 cells were only examined up to 3 days due to the practical constraints of rapidly dividing HepG2 cells in cell culture. After 3 days FIAU induced a dose- and time-dependent increase in cellular ATP content when normalised to cellular protein, with no significant difference between glucose and galactose media (Fig. 2C). Examination of protein content revealed a significant decrease to 49.0 ± 22.7% and 40.5 ± 13.4% of vehicle control of total cellular protein at FIAU concentrations of 300 μM in glucose and galactose media respectively. Overall, these tests show that FIAU cannot be classified as a mitotoxin in HepG2 cells.

IC_50_ values for effects upon cellular ATP content and viability were calculated in order to define whether FIAU was causing cytotoxicity via mitochondrial dysfunction, using the previously described parameters (Table 2) (Kamalian et al., 2015). At week 2 there was a significant difference (*p* 0.03) in IC_50_-ATP_glu_ (16.8 ± 2.3 μM) and IC_50_-ATP_gal_ (7.3 ± 3.8 μM). Applying these parameters, FIAU is shown to be inducing mitochondrial dysfunction after 2 weeks of exposure, with effects on cellular ATP content (ATP/μg protein) preceding effects on cell viability (μg protein). By week 3 the IC_50_ values decreased as FIAU induces greater toxicity and the difference between the ATP dose-response curves in glucose and galactose is lost, although decreases in ATP still precede cell death. The epimer of FIAU, FIAM, was inactive in this assay; no significant differences in cellular ATP content or cell viability compared to vehicle control were measured over the 4-week incubation period, Table 2.

**Table 2:** ATP and viability IC_50_ values (μM) in glucose and galactose media of FIAU and FIAM treated HepaRG cells over a 4-week exposure period. Viability data is derived from total cellular protein measurements of the terminally differentiated HepaRG cells. A mitochondrial perturbation can be identified when IC_50_ATPglu/IC_50_ATPgal is ≥2 with a p value <.05.

	IC_50_-ATP (μM) ± S.D.		IC_50_-viability (μM) ± S.D.		IC_50_ATPglu/IC_50_ATPgal (p value)	IC_50_-viabliity gal/IC_50_ATPgal (*p* value)
	Glucose	Galactose	Glucose	Galactose		
wk 1	112.4 ± 32.8	103.2 ± 13.8	117.5 ± 42.0	100.6 ± 27.0	1.1 (0.69)	1.0 (0.89)
wk 2	16.8 ± 2.3	7.3 ± 3.8	26.1 ± 9.3	7.3 ± 3.8	2.3 (0.03)	1.0 (1)
wk 3	5.4 ± 2.3	5.7 ± 2.8	14.2 ± 1.3	15.5 ± 3.4	0.9 (0.87)	2.7 (0.02)
wk 4	4.9 ± 1.5	7.3 ± 0.5	7.6 ± 3.3	3.6 ± 2.8	0.7 (0.11)	0.5 (0.16)
FIAM WK 4	>600	>600	>600	>600	>600	>600

### 3.3 Fialuridine induces a time- and dose-dependent decrease in mitochondrial DNA copy number in HepaRG cells

**Fig. 4:**
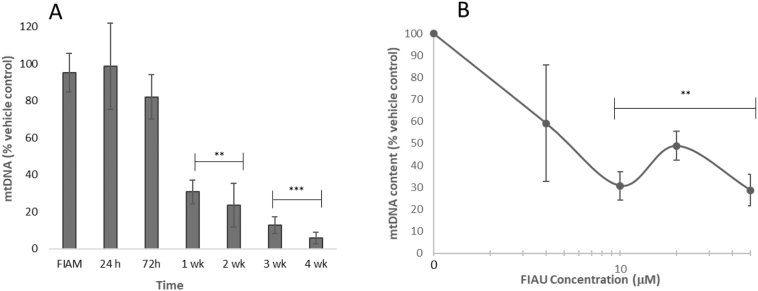
Mitochondrial DNA content in HepaRG exposed to FIAU (0–300 μM, 1–4 weeks). MtDNA content was measured by real-time PCR and reported as a percentage of the vehicle control A: Time-response curve of FIAU following exposure to 10 μM FIAU or FIAM (4 wk). B: Concentration-response curves following 2 weeks exposure to FIAU. Key: Significance of each data point compared to vehicle control; ** p < .01, *** *p* < .001.). *n* = 3.

The effects of FIAU on the amount of mtDNA was assessed in HepaRG cells using real time PCR to quantify mitochondrial copy number normalised against nuclear DNA copy number. FIAU induced a time- and dose-dependent decrease in mitochondrial copy number (Fig. 4). Significant decreases were evident from 1 week of treatment with FIAU (10 μM) where mtDNA content was reduced to 23.5 ± 11.9% of vehicle control, with continued decreases over subsequent weeks reaching a minimum of 5.8 ± 3.0% after 4 weeks (Fig. 4A). Concentration dependency was apparent after 1 week of treatment, with significant decreases evident after 10 μM FIAU, Fig. 4B. In these experiments it was noted that at the concentration dose preceding the significant decrease, measurements were characterised by a large variation in response, see 4 μM FIAU at week 1 (59.2 ± 26.5%, Fig. 4B). The increase in mtDNA observed at 100 μM was not significantly different to its neighbouring points. The same assays were performed with FIAU (1.3–100 μM) in HepG2 cells up to 72 h. However, no significant differences in mitochondrial copy number were observed in these tests (Fig Supp. 3).

### 3.4 Fialuridine induces a decrease in mitochondrial respiration in the absence of dysfunction of the electron transport chain in HepaRG cells

**Fig. 5:**
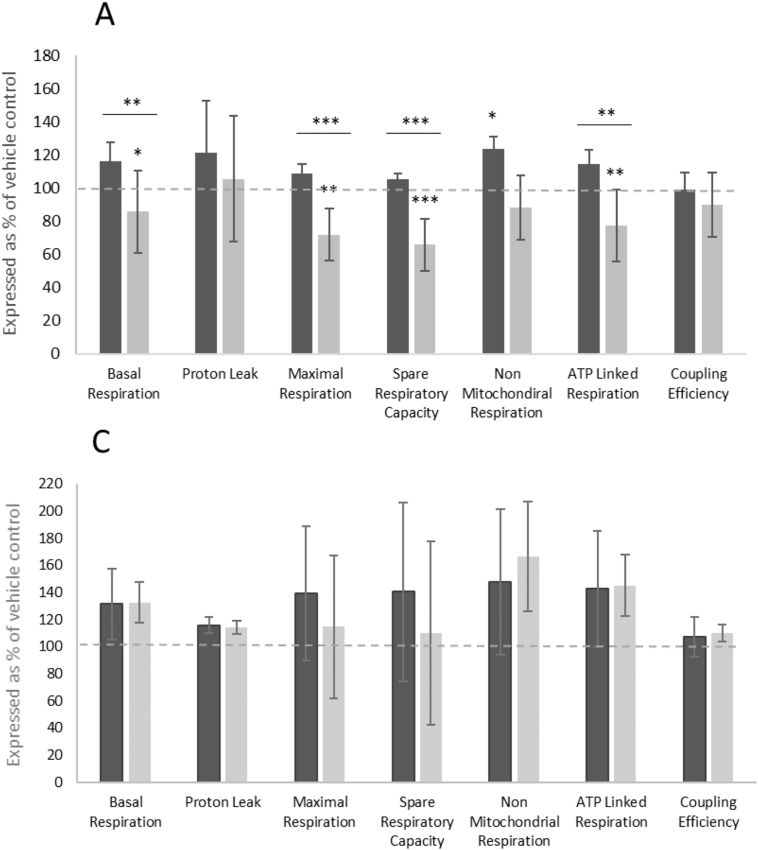
The effect of FIAU (12 μM) on respiratory activity over 2 weeks. A: The bioenergetic parameters were calculated using Seahorse respirometry at week 1 (black bar) and week 2 (grey bar) of FIAU (12 μM) exposure. All results are presented as % of vehicle control. B: Dose-dependent changes in bioenergetic parameters, presented as normalised to maximal respiration, following 2 weeks of exposure to FIAU. C: The bioenergetic parameters of HepaRG cells following exposure to FIAM were calculated following 2 weeks exposure using Seahorse respirometry; 12 μM (black bar) and 100 μM (grey bar). Key: dashed line marks vehicle control at 100%. Significance of each data point compared to either vehicle control or significant difference between week 1 and week 2;*p < .05, ** p < .01, *** p < .001. n = 3.

Mitochondrial respiration was examined using Seahorse Technology. The underlying respiratory usage of oxygen was quantified by performing a mitochondrial stress-test. Specifically, the amount of oxygen consumed for basal respiration (BR), ATP-linked respiration (ALR), maximal respiration (MR), spare respiratory capacity (SRC), proton leak (PL) was measured alongside the calculation of coupling efficiency (CE). A dose- and time-dependent decrease was observed in all parameters of oxygen consumption (Fig. 5). Following 1 weeks of FIAU treatment (12 μM) no significant changes to each parameter were recorded. However, after 2 weeks of exposure several parameters were significantly decreased when compared to vehicle control; BR was decreased to 85.7 ± 28.1%; MR was decreased to 72.0 ± 19.1%; ALR was decreased to 77.4 ± 21.5% and SRC was most changed with a decrease to 65.9 ± 15.5% of vehicle control (Fig. 5A). However, despite overall decreases in basal respiration; PL and CE remained unchanged (Fig. 5A). An absence of significant mitochondrial dysfunction was also noted when calculating the proportion of each of the parameters as a % of MR after 2 weeks of FIAU exposure (Fig. 5B). In this way it can be observed that the proportion of each parameter remains unaltered, indicative of the continued function of the mitochondria despite lower overall respiration. The structural isomer of FIAU, FIAM, did not reduce mitochondrial respiration after 2 weeks when applied at the same concentration (12 μM) and up to a maximal concentration (100 μM). However, several parameters of mitochondrial respiratory function were observed to increase, although these changes were not significant or dose-dependent, notwithstanding PL and CE, which did not change (Fig. 5C). The same assays were performed with FIAU (1.3–100 μM) in HepG2 cells up to 72 h. However, no significant differences in bioenergetic parameters were observed in these tests (Fig Supp 4).

**Fig. 6:**
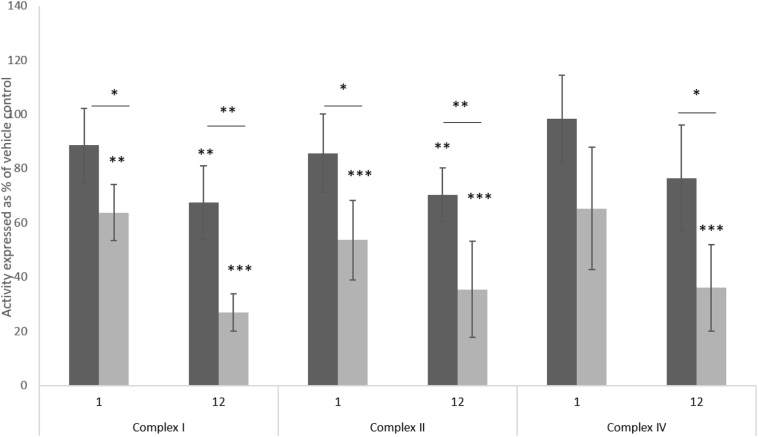
The effect of FIAU (1–12 μM) on individual complex (I, II, IV) activity within the respiratory chain over 2 weeks. The activity of complexes I, II and IV were measured individually in permeabilised HepaRG cells using Seahorse respirometry at week 1 (black bar) and week 2 (grey bar) of exposure to FIAU (1–12 μM) exposure. All results are presented as % of vehicle control. Key: Significance of each data point compared to either vehicle control or significant difference between week 1 and week 2;*p < .05, ** p < .01, *** p < .001. n = 3.

Individual activity of complexes I, II and IV in permeabilised cells was assessed using Seahorse respirometry (Fig. 6). Complex III activity was unable to be accurately assessed due to extreme variability in respiration when using duroquinol as a specific substrate to drive respiration via complex III. Each measurement was normalised to protein content to mitigate against differences in cell number. A significant dose and time-dependent decrease in activity of each complex was observed, with decreases in complex I and II activity becoming significant at the highest doses of FIAU (12 μM, week 1); activities of 67.4 ± 13.5% and 70.3 ± 9.8% of vehicle control respectively. These decreases became significantly greater following 2 weeks of treatment. However, each complex was affected equally by FIAU, with no significant differences measured. For example, FIAU (12 μM) induced a decrease in complex I activity to 26.9 ± 6.9% of the vehicle control; complex II activity to 35.5 ± 17.7% of the vehicle control and complex IV activity to 36.0 ± 15.9% of the vehicle control.

### 3.5 Fialuridine does not induce changes in mitochondria area or number over 2 weeks of exposure

**Fig. 7:**
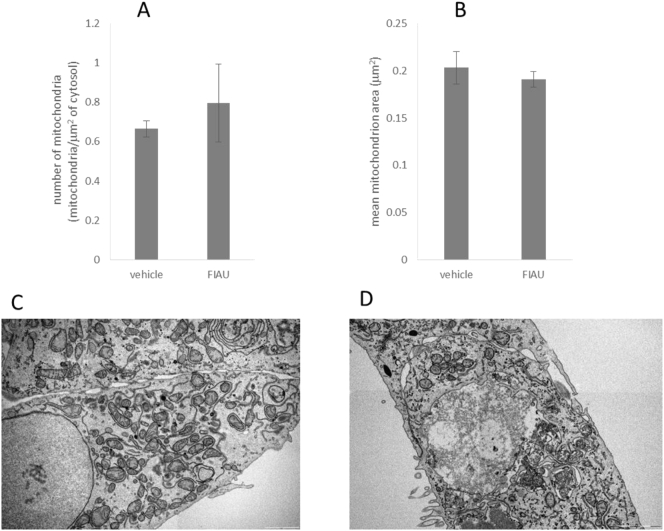
The effect of FIAU on mitochondrial morphology when assessed by electron microscopy over 2 weeks of exposure to FIAU (12 μM). Images produced by electron microscopy were semi-quantitatively assessed for A) number of mitochondria and B) mean mitochondrial area. C) Representative image of HepaRG cells exposed to vehicle control. D) Representative image of HepaRG cells exposed to vehicle control. n = 3.

Several measurements indicate that FIAU has no effect upon mitochondria area or number after up to 2-weeks of treatment, Fig. 7. Electron microscopy images (Fig. 7C, D) revealed that there were no obvious structural mitochondrial abnormalities, changes in mitochondrial density or areas of lipid accumulation when HepaRG cells were treated with FIAU (12 μM, up to 2 weeks) compared to vehicle-treated controls. Semi-quantitative assessment of these images (Figs. 7A, B) revealed that there were no significant differences in individual mean mitochondrial area (vehicle-treated cells; 0.20 ± 0.02 μm^2^ versus FIAU-treated cells; 0.19 ± 0.01 μm^2^) or number of mitochondria (vehicle-treated cells; 0.66 ± 0.04 mitochondria/μm^2^ of cytosol versus FIAU-treated cells; 0.80 ± 0.20 mitochondria/μm^2^ of cytosol). The lack of any differences between vehicle and FIAU treated cells when examined by EM was observed in tandem with no apparent change in mitochondrial mass when assessed by western blot assessment of VDAC, a structural mitochondrial protein, which stayed constant across a dose and time-range indicative of no change in mitochondrial mass following FIAU treatment (data not shown).

### 3.6 There is no evidence of steatosis or lactic acidosis in vitro over 4 weeks of fialuridine treatment

The accumulation of intracellular lipids was examined after 4 weeks of FIAU (12 μM by oil red O staining. There was no accumulation of intracellular lipids over this time-course, which coincides with an absence of lipid droplets when examined by EM at 2 weeks. Chloroquine (72 h, 30 μM) was included as a positive control (data not shown).

Extracellular L-lactate concentrations were monitored over a four-week exposure to FIAU (12 μM). There was no significant increase in L-lactate in the cell supernatant (data not shown). In addition, the extracellular acidification rate was monitored in real-time using Seahorse technology. Over the 2 weeks of FIAU treatment there were no dose- or time-dependent increases in the extracellular acidification rate (mpH/min/mg protein), a rate which reflects the change in pH from both glycolytic and oxidative phosphorylation proton production. This is an indication that extracellular acidification was not induced by FIAU in HepaRG cells.

## 4 Discussion

In previous pre-clinical testing the failure to predict FIAU induced hepatotoxicity in patients was largely due to the in vitro and in vivo test systems lacking the correct physiological and pharmacological phenotype. Here, proteomic analysis confirms that both HepG2 and HepaRG cells possess the correct physiological characteristics, specifically the presence of hENT-1, TK-1, TK-2 and thymidylate kinase, with which to study the toxicological effects of FIAU. Interestingly, HepaRG have lower levels of the critical hENT1 transporter, than HepG2, which suggests that the lack of effect of FIAU over 72 h observed in this study does not occur due to a lack of entry to the mitochondria. However, the ability of HepaRG cells to remain differentiated in culture over 4 weeks give the model a clear advantage when studying the effects of longer term, clinically relevant FIAU exposure and a delayed onset of toxicity. The analysis also suggests the presence of intra-individual differences across the 3 cryopreserved PHH samples which may be a limitation of the use of PHH, fresh or cryopreserved, for drug toxicity studies of this kind. It should be noted that rodent hepatocytes could not be adopted as an alternative fresh, primary system with less variability, due to the specificity of the hENT1 transporter to the mitochondrial membrane only in humans or chimeric humanised mouse models (Xu et al., 2014).

The acute modification assay is based on “switching” sugar sources in media from glucose to galactose to reveal mitochondrial toxicity that can be obscured by the Crabtree effect. In brief, replacing glucose with galactose does not prevent glycolytic ATP-production, instead the conversion of galactose to glucose consumes ATP resulting in a near-zero net production of ATP from glycolysis, thus exposing previously masked dysfunction of oxidative phosphorylation (Marroquin et al., 2007). Here, the assay was adapted for use over extended dosing periods so that the sugar switch, or “metabolic modification”, was only performed for the final 2 h of incubation. This short switch is possible because the presence of glucose or galactose does not affect the induction of mitochondrial dysfunction, instead, galactose is required only to reveal subsequent effects upon ATP production. Testing revealed that the effect of FIAU on HepaRG cells was mediated via mitochondrial dysfunction as IC_50_-ATPglu/IC_50_-ATPgal ≥2, following 2 weeks of exposure. Importantly, FIAM did not induce any decrease over 4 weeks, indicating that these effects are a consequence of the structural pharmacophore of FIAU.

The published Cmax of FIAU in healthy volunteers is 0.6 μM, measured following a 5 mg dose single dose (Bowsher et al., 1994). This can be used to estimate a Cmax in patients experiencing DILI, which was not measured, to be approximately 2–2.5 μM based upon a dosing range of 0.1–0.25 mg/kg/day. Therefore, the effects reported here, IC_50_-ATP gal ~16 μM following 2 weeks of exposure, decreasing to 5 μM over the next 7 days, are within a range of 10–15 X Cmax and can be deemed to have clinical relevance. In addition, the repeated administration of the test compounds every 3–4 days is closer to the repeated doses administered in the clinic. In related research, FIAU toxicity was assessed in a 3D model of PHH spheroids (Bell et al., 2016), which demonstrated that when in 3D, PHH have increased sensitivity to FIAU, quoting an IC_50_-ATP of 0.7 μM after 7 days. Subsequent research indicated that in 3D PHH culture, toxicity precedes via effects on mitochondrial DNA (Hendriks et al., 2019).

It has previously been reported that FIAU induces toxicity in HepG2 cells; a TC_50_ (Toxicity Concentration) of 35 μM following 6 days of treatment (Colacino et al., 1994), in contrast to the current report in where very little effect was seen following a 3 day exposure. However, in the current assays it is important to note that cellular ATP does not reflect changes in cell number; cellular ATP content is expressed per μg of cell protein. In fact, further examination revealed a decrease in protein content which was not due to necrotic cell death, determined using cellular LDH release (data not shown). Overall, this suggests that in HepG2 cells FIAU may have anti-proliferative effects, which is in line with the aforementioned study by Colacino et al. where cytotoxicity data was based upon cell number. This important difference between effects on proliferation and cell death, alongside a variation in exposure time, unifies several other studies which report the absence of cytotoxicity induced by FIAU in HepG2 cells (Lewis et al., 1996; Birkus et al., 2003). It is known that the effects of mtDNA disruption on cell health can take weeks to accumulate to a level at which cellular toxicity is observed. This effect is based upon a need for mitochondrial damage to accumulate to a certain level, estimated to be 60–90% of function, termed the “threshold effect” before cellular energy production no longer supports cell viability and cell toxicity is triggered (Rossignol et al., 2003). This ability to preserve energy production is made possible by the multiplicity of mitochondria in each cell (100 – 1000s) and its heteroplasmic distribution, alongside the presence of several compensatory energy production pathways and active mitochondrial quality control processes which can be initiated in times of respiratory stress, including fusion, fission and mitophagy. Thus, the differentiated nature of HepaRG cells may allow for the accumulation of mitochondrial dysfunction, becoming measurable at 2 weeks, which is not possible in a standard proliferating cell line which depends on mitochondrial respiration to support continued growth. The reported experiments do not define the pathway cell death initiated in HepaRG following the inhibition of mitochondrial replication and subsequent decrease in mitochondrial respiration. Based upon changes in ATP levels, oncosis and apoptosis are two such candidate pathways (D'Arcy, 2019). Specifically, apoptosis is an active process which and so requires ATP and oncosis, which is characterised by a rapid decrease in intracellular ATP levels preceding cell swelling are both associated with fulminant liver toxicity (Luedde et al., 2014). Although, a decrease in ATP levels were observed, it is not clear without further experimentation whether these were a cause of subsequent cell death, such as in oncosis, or a result of cell death such as apoptosis. No further morphological indication of pathway was noted during electron microscopy studies and therefore further biochemical experimentation would be required.

FIAU-induced mitochondrial DNA depletion is concentration-dependent, beginning at 72 h, becoming significant after 2 weeks at concentrations approximately equal to the IC_50_. This is a clear indication that in HepaRG cells FIAU is inhibiting mtDNA replication at clinically relevant concentrations. Interestingly, a previous study failed to observe any inhibition of mtDNA replication in HepG2 cells (6 days), although a separate study did report decreases following 9 days of FIAU exposure but at high concentrations (30 μM)(Colacino et al., 1994). This result was replicated in the present study; mitochondrial copy number was not decreased in HepG2 cells over the time course. Many drugs in the NRTI class are known to dysregulate mitochondrial function, however, the precise interaction with this process can vary depending on NRTI structure. Phosphorylated NRTIs, including FIAU, can be incorporated into the mtDNA chain at adenosine sites due to their nucleoside-based structure (Lewis et al., 1996), leading to the well-known NRTI initiated inhibition of the mitochondrial DNA polymerase γ (Cherrington et al., 1994; Lewis et al., 1994). With most NRTI's such incorporation results in a rapid termination of mtDNA chain extension. However, FIAU has a hydroxyl group at the 3′ position which allows continued DNA chain extension. This structural similarity to native nucleotides is hypothesised to be the reason for its extreme toxicity as the DNA machinery is unable to recognise a FIAU insertion and inhibition of chain elongation only occurs at sites of multiple insertion. Thus, the delayed effects on mtDNA replication observed here match with this slower accumulation of mitochondrial damage by FIAU in the absence of chain termination.

Next, for the first time, the functional consequences of the inhibition of mtDNA replication upon oxidative phosphorylation were defined. Respirometric analysis demonstrated that the earliest, and most significant, change was a decrease in SRC, a change previously described as a warning flag for mitochondrial dysfunction (Kamalian et al., 2018). Subsequent to this a decrease in ALR was observed, in line with the decrease in cellular ATP-content observed. Using the framework postulated by Kamalian et al. for the mechanistic interpretation of Seahorse data, a decrease in ALR and SRC occurring in tandem with a decrease in BR indicates that FIAU inhibits activity of the electron transport chain (Whitaker et al., 2015; Ball et al., 2016; Kamalian et al., 2018). Further analysis offers additional mechanistic insight. Specifically, despite a decrease in respirometric activity there is no evidence that the ETC is uncoupled or damaged, as no changes in PL and CE were observed in concert with an unchanged relative proportion of oxygen consumption linked to ALR, SRC and PL. Overall, these results suggest that an alternative effect on the mitochondria is induced in which total mitochondrial respiratory activity is “turned down/reduced” as opposed to dysfunction of specific components, hence CE and PL remain constant. This view is supported by the dose- and time-dependent reduction in the activity of the individual complexes within the ETC. Importantly, this decrease in oxidative phosphorylation and complex activity is associated with any loss in mitochondrial mass or number. Taken together these results are suggestive that the effect of FIAU upon respiration results from a reduction in the translation of proteins encoded by the mitochondrial genome, i.e. thirteen electron transport chain subunits spanning CI, III, IV and V, which results in a reduction in complex activity and subsequent decrease in ETC without signs of dysfunction. The concomitant reduction in activity of wholly nuclear-encoded CII may arise due to mitochondrial retrograde signalling as a homeostatic response to a down-regulation in OXPHOS activity or due to a subsequent inhibition of mitochondrial biogenesis (Cardamone et al., 2018). As the complexity of the pathways controlling and coordinating mitochondrial gene expression, protein translation and respiratory function becomes more evident (Pearce et al., 2017; Cardamone et al., 2018), the FIAU-HepaRG model should be viewed as a platform from which to understand and delineate these mechanisms more clearly.

The pathological features previously reported in affected patients were not observed in the HepaRG model of FIAU toxicity*.* Specifically, lactic acidosis; the onset of steatosis and the presence of morphologically abnormal mitochondria. It may be that the shortened time of onset of toxicity, specifically by 2 weeks in vitro, the effects of FIAU can be observed at the mitochondria and cellular levels. However, in patients the onset of gross morphological and clinical signals were not observed until week 13, perhaps due to some adaptive or protective mechanisms not recapitulated in a simple cellular model. In addition, the affected patients had already been exposed to FIAU in previous, shorter clinical trials which may also contribute to this difference.

Overall, these investigations have demonstrated for the first time that the differentiated nature of HepaRG cells allow the modelling of delayed onset FIAU-induced toxicity, which is not possible in the standard, proliferating HepG2 cells in which the inhibition of mtDNA replication leading to mitochondrial dysfunction and cell death were not observed over a short 72 h period. The extended exposure period afforded by the HepaRG model allowed the temporal elucidation of mitochondrial toxicity pathways which begins with an early effect upon mtDNA and bioenergetic function, visible after 1 week of treatment and subsequently leads to decreases in mitochondrial respiratory function and the induction of cell death after 2 weeks of repeated exposure. This lag period between the first point of mitochondrial insult and cell death is reflective of the delayed onset seen in the failed clinical trial. The findings of this research are supported by a recent study which aimed to use the HepaRG model to evaluate the effect of nine hepatotoxins upon mtDNA and cytotoxicity following extended exposure (Le Guillou et al., 2018). Although the FIAU clinical trial incident occurred over 20 years ago, it remains highly relevant to drug development today and despite significant advances in understanding the mechanisms of FIAU-induced toxicity it remains unlikely that traditional preclinical safety screening over short periods would fully predict FIAU hepatotoxicity. However, the approach described in this research to ensure that phenotype of the model system is fit for purpose with respect to both the drug- and patient-specific factors has led to the development of a model which is able to reproduce concentration and time-dependent mitochondrial and cellular toxicity at clinically relevant concentrations. Such mechanistic information would have been critical in the preclinical safety evaluation of FIAU, and may have relevance for other, novel agents in the class of nucleotide-based antiviral therapy. For example, the relevance of assessing investigational drugs on mitochondrial toxicity in proliferating cell lines (e.g. mitochondrial DNA content, lactic acid production, glucose utilization) was acknowledged in the FDA's Guidance for Industry (Nov 2015) “HIV-1 infection: developing antiretroviral drugs for treatment”. Drug classes which should be evaluated for mitochondrial toxicity in proliferating cell lines over a prolonged incubation period include nucleoside drugs forming triphosphate active metabolites and oligonucleotide drugs like siRNAs which after nuclease metabolism can form monomers which can be phosphorylated by kinases to triphosphate metabolites. The phosphate metabolites can interfere with deoxyribonucleic acid (DNA) polymerases and mitochondrial DNA. Although it is important to note that the results reported herein arise from the study of one compound with a specific mechanism, and are not exhaustive, they indicate that this system may find utility in the investigation of mitochondrial toxicity over extended period has utility for many other mitochondrial toxins which manifest delayed toxicity which for which in vivo models are not available. This opportunity should be the focus of future work.

## Funding

This work is supported by the European Community under the 10.13039/501100010767Innovative Medicines Initiative (IMI) Programme through [Grant Agreement number 115336 (MIP-DILI)] and Janssen Pharmaceutica N.V. [agreement ICD #387882], as part of the Drug Safety Centre supported by the 10.13039/501100000265Medical Research Council [grant number G0700654] to [LK]. [OD] was funded by an MRC Case Award in collaboration with AstraZeneca.

## Declaration of Competing Interest

The authors declare that they have no known competing financial interests or personal relationships that could have appeared to influence the work reported in this paper.

## Appendix A Supplementary data

Supplementary material Image 1
