# Supplementary material for: The utility of a differentiated preclinical liver model, HepaRG cells, in investigating delayed toxicity via inhibition of mitochondrial-replication induced by fialuridine
Source: Toxicol Appl Pharmacol. 2020 Sep 15;403:115163. doi: 10.1016/j.taap.2020.115163 (PMC7456776; doi:10.1016/j.taap.2020.115163)

SUPPLEMENTARY FIGURES

Figure Supp 1: Representative image of differentiated HepaRG cells. HepaRG cells are differentiated in situ, over 4 weeks, to hepatocytes and biliary-like epithelial cells. Key: A, Hepatocyte clusters and B, biliary epithelial-like cells.


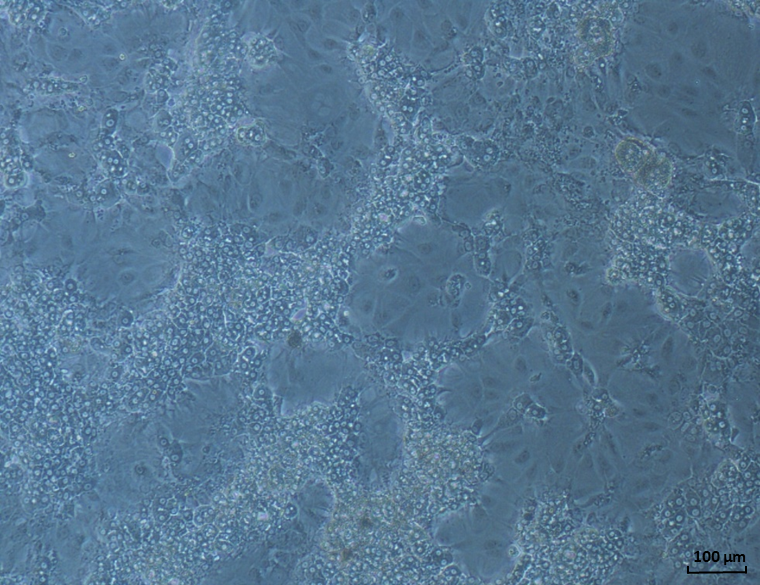


**A**

**B**

**A**

**B**

Figure Supp 2 The proteome of HepaRG cells was compared to that of primary human hepatocytes using iTRAQ analysis. Mass spectral evidence for the presence of proteins involved in mitochondrial uptake of fialuridine was extracted from the complete dataset. The sequence coverage (99% confident identified peptides shown in green) and representative MS/MS spectra are shown for A. hENT-1, B. thymidylate kinase, C. TK1 and D. TK2


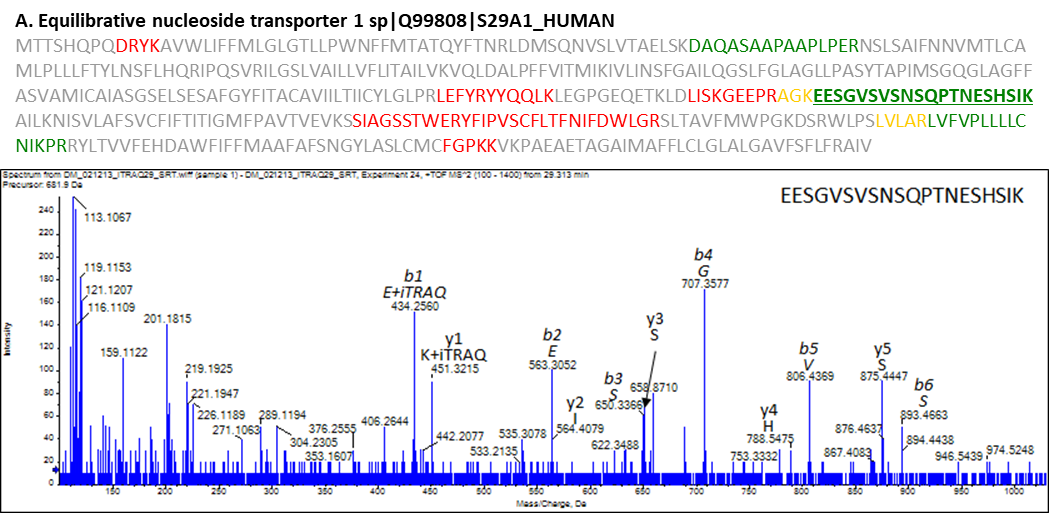


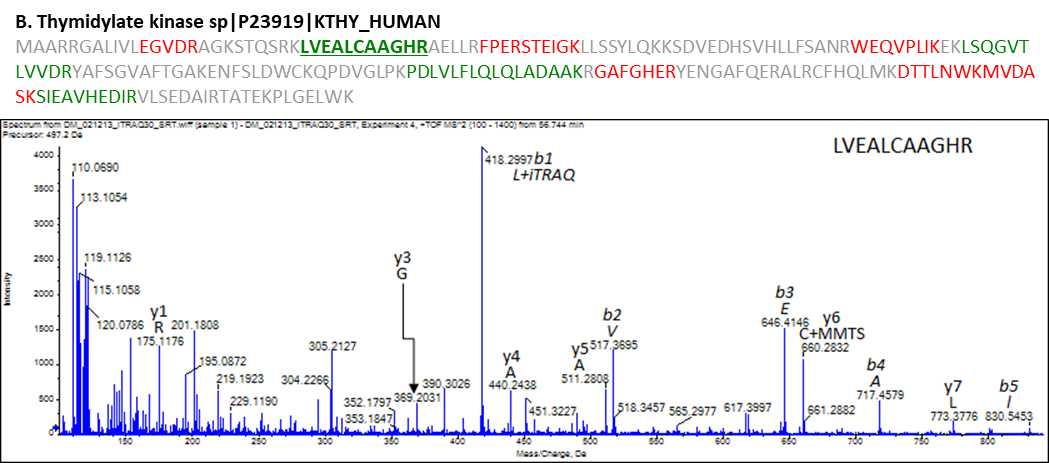


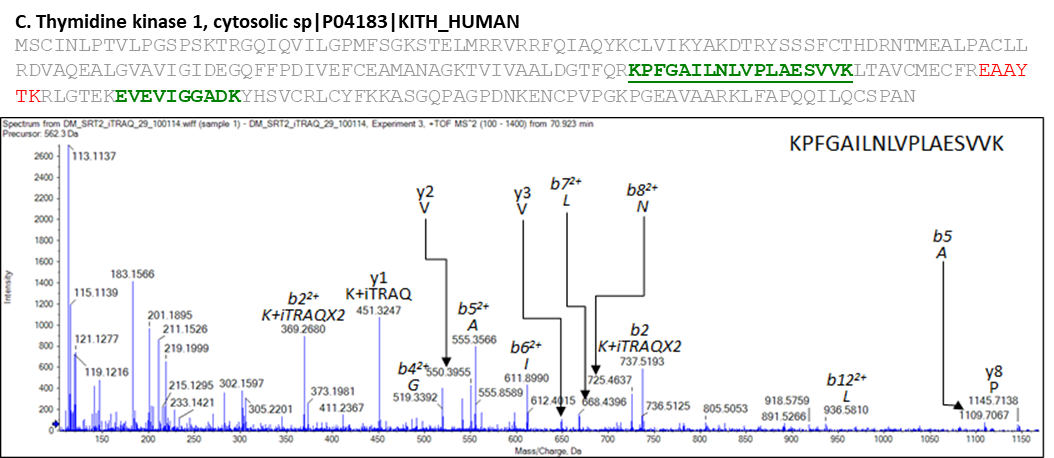


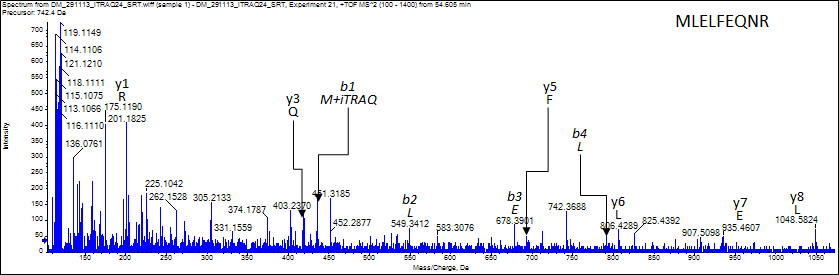


**D. Thymidine kinase 2, mitochondrial sp|O00142|KITM_HUMAN**

MLLWPLRGWAARALRCFGPGSRGSPASGPGPRRVQRRAWPPDKEQEKEKKSVICVEGNIASGKTTCLEFFSNATDVEVLTEPVSKWRNVRGHNPLGLMYHDASRWGLTLQTYVQLTMLDRHTRPQVSSVRLMERSIHSARYIFVENLYRSGKMPEVDYVVLSEWFDWILRNMDVSVDLIVYLRTNPETCYQRLKKRCREEEKVIPLEYLEAIHHLHEEWLIKGSLFPMAAPVLVIEADHHMER**MLELFEQNR**DRILTPENRKHCP

Figure Supp 3: Mitochondrial DNA content in HepG2 cells exposed to FIAU (48 and 72 h). MtDNA content was measured by real-time PCR. HepG2 cells were exposed to FIAU (10 and 100 µM) at 48 h (black bar) and 72 h (grey bar). All results are presented as % of vehicle control. n=3.

Figure Supp 4: The effect of FIAU on mitochondrial respiratory activity of HepG2 cells. The bioenergetic parameters of HepG2 cells following exposure to FIAU (12 µM) were calculated using Seahorse respirometry at 48 h (black bar) and 72 h (grey bar). All results are presented as % of vehicle control. Key: dashed line marks vehicle control at 100 %. n=3.


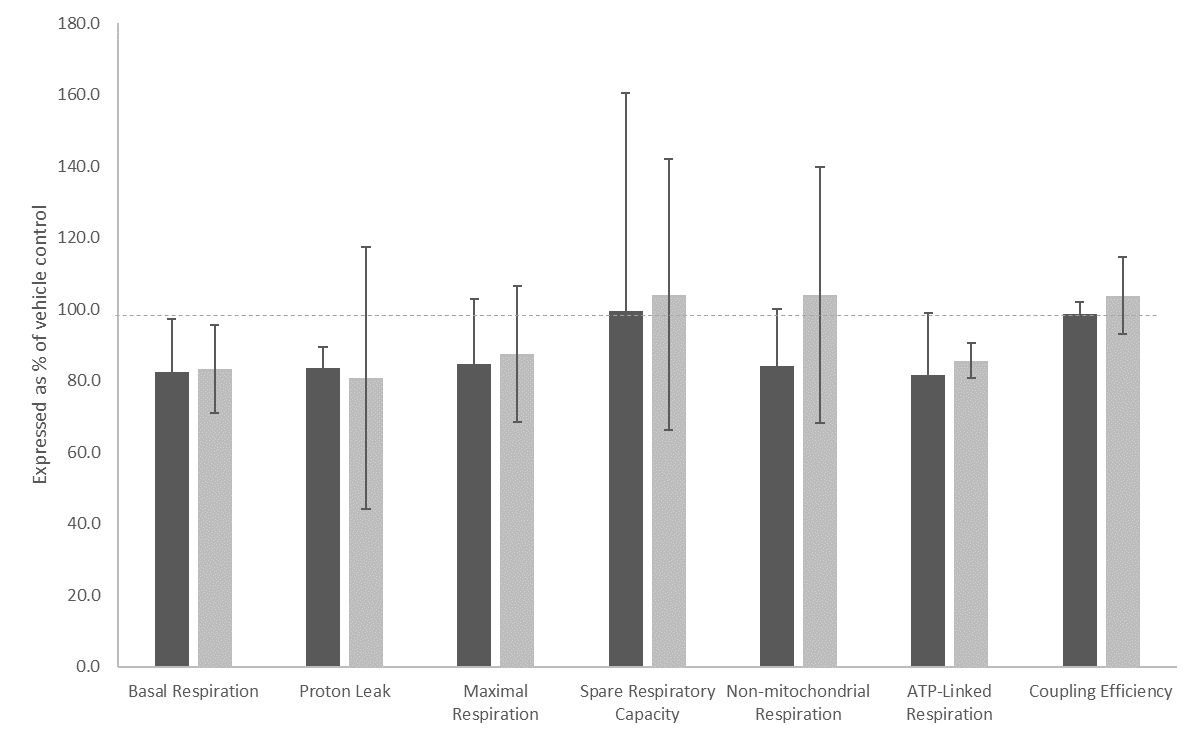

Supplement: Supplementary file 1 — Supplementary material [file mmc1.docx]
